# Family processes and structure: Longitudinal influences on adolescent disruptive and internalizing behaviors

**DOI:** 10.1111/fare.12728

**Published:** 2022-07-06

**Authors:** Shannon M. Savell, Ravjot Saini, Mayra Ramos, Melvin N. Wilson, Kathryn Lemery‐Chalfant, Daniel S. Shaw

**Affiliations:** ^1^ Department of Psychology University of Virginia Charlottesville VA; ^2^ Department of Psychology Arizona State University Tempe AZ; ^3^ Department of Psychology University of Pittsburgh Pittsburgh PA

**Keywords:** adolescent disruptive behavior, adolescent internalizing problem behavior, family process, family structure, longitudinal prospective design

## Abstract

**Objective:**

The present study revisits the assumption in American culture, based in “family privilege,” that children fare better in two‐parent households by longitudinally examining associations between family structure, process, and adolescent behavior.

**Background:**

Societal assumptions and cross‐sectional research suggest that there is a difference in child adjustment across varying family structures. Relatedly, the family process literature emphasizes the importance of parent–child relationship quality in addition to family structure on child adjustment.

**Method:**

We utilized a longitudinal, prospective design that assessed family structures on nine occasions covering a 12‐year period beginning when the target child was 2 years of age for a large (*N* = 714), ethnically and racially diverse sample of low‐income families. We examined the relation between self‐reported, teacher‐reported, and primary caregiver‐reported adolescent disruptive and internalizing problem behavior across family structures and parent–child relationship quality.

**Results:**

Across seven identified family structures, adolescent behavior did not differ after accounting for middle‐childhood adjustment and relevant contextual factors. However, consistent with family process models of child adjustment, positive parent–child relationship quality predicted lower rates of adolescent maladaptive behavior.

**Conclusion:**

These findings serve to combat stigma related to family structures that deviate from married parents raising their children and highlight the need for interventions designed to foster positive parent–child relationships.

**Implications:**

Policy makers and practitioners should aim to support efforts to foster positive parent–child relationships across types of family structures and refrain from promoting or discouraging the formations of specific family structure types.

Based on the diversity in family structures present in American society today, it is important to interrogate assumptions about the impact on child behavior that varying types of family structures may have. A vast majority of the previous literature has compared children of married biological parents with those of divorced parents (Amato, 2010). Decades of research on the impact of divorce on child behavior (for review, see Shaw, 1991; Ganong & Coleman, 2018) would lead one to assume there would be significant differences across different family structures in child adjustment, including among low‐income families that disproportionately include larger percentages of single‐parent families that often do not have the additional income and resources that two‐parent households offer (Musick & Meier, 2010).

Many scholars have recently called attention to the power and privilege afforded to certain family structures (e.g., nuclear families; Ganong et al., 2015; Letiecq, 2019). To that end, Russell et al. (2018) argued that family structure may be a social determinant of health based on the social stratification across family structure types. The influence of “family privilege,” as noted by Letiecq (2019), has been evident in social science research, particularly in research on family structure in which generalizations about the impact of various family structures on many aspects of child development have been drawn from relatively little evidence or without proper acknowledgement of study limitations (Ganong & Coleman, 2018). Privileging and centering certain family structures is evident not just in research practices but also in public policy. For example, over the past few decades, the United States policy agenda has encouraged two‐parent households by promoting increases in marriage rates as a tool for improving child outcomes (Nock, 2005). The present study interrogates assumptions steeped in family privilege about diverse family structures and examines whether adolescent internalizing and disruptive behaviors are similar across manifold family structures over the course of 12 years of child development in a large (*N* = 714), racially diverse sample of low‐income families.

## The family context and child adjustment: Family structure

The family context is considered one of the most influential environments for child development (Bronfenbrenner, 1986). Bronfenbrenner's ecological systems theory (1986) highlights the importance of family context on child behavior and development as a microsystem level influence. Structure and processes, both facets of the family context, influence children's behavior and development in different ways. Traditionally in the literature, research on the impact of family structure on child prosocial and problem behavior has been conducted by comparing child adjustment across different types of family structure (e.g., married biological parents versus single‐parent families; see Beckmeyer & Russell, 2018; Krueger et al., 2015). Developmental outcomes in the family structure literature have focused on adolescent academic and social outcomes as well as depressive and anxious symptoms, with less attention devoted to disruptive behaviors in adolescence (Langton & Berger, 2011). Thus, in addition to examining internalizing problem behavior, we focus on disruptive behaviors, as reported by adolescents, parents, and teachers to assess child disruptive behavior within and outside of the home.

Previous research suggests that there is a strong association between family structure and adolescent behavioral and health problems (Dunifon & Kowaleski‐Jones, 2002; Magnuson & Berger, 2009). However, many of the previous studies in this area utilized a cross‐sectional design, used only one informant, or focused on only one developmental period. Interestingly, the studies that have examined family structure and child behavior cross‐sectionally show contradictory results (Hadfield et al., 2018). For example, Magnuson and Berger (2009) found that two‐parent biological family structures had larger declines in their children's behavioral problems, as reported by their mothers, from age 6 to 12 years in comparison to stable single‐mother family structures, after accounting for mean family income‐to‐needs and maternal employment. Similarly, Dunifon and Kowaleski‐Jones (2002) found that for African American early adolescents (10‐ to 14‐year‐olds), self‐reported disruptive problem behaviors were greater in cohabiting family structures than two‐parent biological family structures. Furthermore, European American early adolescents had lower math scores in cohabiting family structures after accounting for average family income, number of children in the household, and maternal employment. As many scholars have noted, the influence of family structure on certain childhood outcomes (e.g., education; Cross, 2020) operates differently across various cultural and racial backgrounds of families, which may contribute to mixed findings in the literature.

However, other studies have found no significant differences between children or adolescents in certain types of family structures. Using a large, nationally representative sample of families and their adolescents, DeLeire and Kalil (2002) found that adolescents living with their single mothers and their grandparent(s) in multigenerational households had college attendance rates, substance use rates, and age of sexual debut on par with youth from married families when accounting for economic resources, parenting behavior, and home and school characteristics. Similarly, Manning and Lamb (2003) found no evidence to suggest that stepfather presence, regardless of whether cohabitating or married, was related to adolescent delinquency or academic achievement when compared to single‐parent families.

From a family systems theory framework, family structure is thought to influence adolescent internalizing and disruptive behaviors because of differences in parental resources and parenting stress across different family structures (Beckmeyer et al., 2020; Demuth & Brown, 2004; Pettit et al., 2007). One finding consistently shown in the current literature is that the single‐parent family structure, which tends to be associated with higher levels of parenting stress (Berryhill, 2016), is also associated with children's heightened risk for maladaptive development when compared to two‐parent households; however, a significant limitation of the findings may be that single‐parent households generally earn lower incomes and have fewer resources compared with two‐parent households (Ryan & Claessens, 2013). As suggested by Brown (2004), a potential mediating variable for the increased risk associated with single‐parent family structures may be socioeconomic conditions that result from a dissolution of a relationship and change in the family structure. Further, socioeconomic conditions may differ in single‐parent households in which the parent never married or cohabitated with a partner, which is a growing family structure in modern American society (Gray et al., 2016). The current study utilized a sample that was comparably economically disadvantaged at recruitment, with individual differences in family income accounted for in the analyses.

With a few exceptions (e.g., Block et al., 1986; Cherlin et al., 1991; Shaw et al., 1993; Shaw et al., 1999), most of the previous longitudinal work in this area has focused on child behavior following parental separation. Because of the biases inherent in retrospective reporting, divorced parents may blame children's problematic behavior on the divorce rather than circumstances that existed prior to the parental separation (Shaw et al., 1999). Prospective examinations of associations between adolescent behavior and modern family structures across multiple developmental time periods are critically needed to extend the current literature.

## Family context and child behavior: Family process

An area of the literature that emerged from findings on the impact of divorce on children is investigating the importance of family *process* rather than family *structure* on child behavior outcomes (Acock & Demo, 1994; Emery, 1982; Ganong & Coleman, 2018). Among others, family processes include parent–child relationship quality, caregiver romantic satisfaction, parenting practices, and parent mental health (Bronfenbrenner, 1986; Walsh, 1982). A strong parent–child relationship, for example, is associated with a myriad of positive outcomes for children (Sarsour et al., 2011) and adolescents (Hines, 1997). Children with a positive parent–child relationship are more likely to have positive peer relationships and positive social outcomes in school in late childhood and early adolescence (Ketsetzis et al., 1998). Additionally, parent support and relationship quality are associated with high levels of academic achievement in early adolescence (Morrison et al., 2003) and academic motivation throughout adolescence (Nuttall & Nuttall, 1976). Further, a positive parent–child relationship has been shown to attenuate rates of adolescent risk‐taking behaviors (Qu et al., 2015) and adolescent depressive symptoms (Branje et al., 2010). However, studies that prospectively assess parent–child relationship quality across multiple developmental periods and their associations with adolescent adjustment are not well‐represented in the literature. Positive parent–child relationship quality has also been shown to act as a buffer against risk for developing disruptive behaviors in adolescence typically associated with children's experience of adverse events (O'Donnell et al., 2002; Savell et al., 2019). Further, positive parent–child relationship quality is associated with lower rates of adolescent problem behaviors (Rothbaum & Weisz, 1994), even though adolescence is a developmental period known for greater autonomy, more time spent outside of the home, and an increase in the influence of the quality of peer relationships (Côté, 2009).

## Recent developments in family structure in the United States

There are a number of gaps in the current literature on family structure and child behavior, namely, its focus on White, middle‐class families and married couples raising their biological children (Amato, 2010). Although the divorce rate in the United States has declined since the 1980s, there has been an increase in the proportion of children born to unmarried, cohabiting parents (Bianchi & Casper, 2000; Kennedy & Bumpass, 2008; U.S. Census Bureau, 2001). Consequently, researchers need to reexamine previous work to incorporate the growing forms of modern family structure. More than 40 years ago, Kellam et al. (1977) seminal work distilled 86 family types found in one urban sample down to 10 major classes of family structure in similarly economically disadvantaged families as the current sample, finding that children of single mothers, as compared to mother–grandmother or mother–stepfather parenting dyads, were most at risk for behavioral problems in early childhood.

More recent research on family structure has included cohabitating biological and stepparent dyads as well as single parents and their relatives raising children in intergenerational family structures (Dunifon & Kowaleski‐Jones, 2007; Mollborn et al., 2011; Murry & Lippold, 2018). However, structures that include single parents who are dating or with relatives living in the home have not been well represented in the family structure literature despite being relatively common in low‐income families (Gray et al., 2016; Hadfield & Ungar, 2018). Few studies to date have assessed differences in adolescent behavior across such diverse modern family structures all in one sample beginning in early childhood and followed prospectively through mid‐adolescence.

## Current study

We sought to investigate the importance of the constructs of family *process* and family *structure* on self, parent, and teacher reports of adolescent problem behavior outcomes. Our first aim was to address associations between family structure and adolescent disruptive and internalizing behavior. Family structure is especially poised to influence adolescent disruptive and internalizing problem behaviors because parenting resources and parenting stress, both established predictors of problem behavior, have been shown to differ across family structures (Amato, 1993; Amato, 2001; Thornton, 2001).

Heeding the call by Powell et al. (2016) and others (e.g., Jensen, 2021) to refrain from centering and privileging two‐parent families as a paragon, the present study examines associations among family structure types and youth maladaptive behavior that extend beyond the two‐parent versus one‐parent comparisons. Although previous studies of mostly middle‐class White children show negative effects for family structures that deviate from those privileged in American culture on child behavior, varying family structures are more normative in low‐income families (Gray et al., 2016) and, consequently, adolescent behavior may be more thoroughly accounted for by other child, family, and community factors beyond family structure type. Therefore, even though there may be differences across family structure types for child behavior outcomes, those differences may be attenuated by relevant covarying factors.

Further, in line with recent work (e.g., Beckmeyer & Russell, 2018; Ganong & Coleman, 2018), we also sought to examine the influence of family process while accounting for family structure type on adolescent problem behavior, as it is possible that the quality of family processes rather than the family structure type may be more strongly associated with adolescent problem behavior. As one index of family process consistently linked to adolescent problem behavior across family structures and socioeconomic status (SES) is *parent–child relationship quality* (Beckmeyer & Russell, 2018; Branje et al., 2010; Demo & Acock, 1996), we expected that a positive parent–child relationship quality from early childhood through adolescence, characterized by lower levels of conflict and higher levels of warmth, support, and communication, would be significantly associated with lower levels of reported adolescent disruptive and internalizing problem behavior.

Using a longitudinal, prospective design that assessed family structure on nine occasions covering a 12‐year period beginning at target child age 2 years, we compared children's teacher‐reported, primary caregiver (PC)–reported, as well as adolescent self‐reported disruptive and internalizing problem behaviors across seven relatively commonly found family structures. In addition to PC reports, we utilize teacher reports and self‐reports of adolescent behavior to reduce the potential for reporter bias in the associations between family structure, family processes, and adolescent behavior. Additionally, we account for more proximal child behavior by including age 10.5 reports of disruptive and internalizing problem behavior as covariates in analyses to reflect possible change in behavior from middle childhood to adolescence. The family structures varied based on the PC having a romantic partner, whether the partner remained living in the home, whether the PCs were romantically involved or were relatives, and whether the PC remained the same over time. We utilized a similar approach in identifying family structure types in the sample to previous researchers (e.g., Lee & McLanahan, 2015; DeLeire & Kalil, 2002) who were also using large, longitudinal data sets of low‐income families (e.g., the Fragile Families Project), emphasizing consistency in PC status over time and relationship of the PC to the child. Importantly, we investigate these research questions in a sample of low‐income families from diverse racial and ethnic backgrounds, unfortunately, a population underexamined in the current research literature on family structure and processes.

## METHODS

### Participants

The ongoing longitudinal Early Steps Multisite project has 731 participating families. Primary caregiver–child dyads were initially recruited when the child was between 2 years 0 months and 2 years 11 months of age from the Special Supplemental Nutrition Program for Women, Infants, and Children (WIC) in the metropolitan areas of Pittsburgh, PA, and Eugene, OR, and the city of Charlottesville, VA, and surrounding counties (Dishion et al., 2008). The PC was the main adult provider of childcare, generally the biological mother of the target child (TC) but could also be another adult caregiver such as the grandparent or other nonrelative. The PC also designated an alternate caregiver (AC), who could be any significant adult that was recognized by the TC and PC and in most cases was the father figure. Inclusion criteria for recruitment included risk factors within the following three domains: (a) child behavior (e.g., conduct problems), (b) family problems (i.e., maternal depression, substance‐use problems), and (c) sociodemographic risk (i.e., no more than 2 years' post–high school education and low family income).

The sample consisted of children who exhibited at least two out of the three risk factors, but all children in the sample had above‐normative levels of externalizing problems to increase parent's motivation for concern. Half of the sample was randomly assigned to the Family Check‐Up intervention condition, which focused on supporting families in developing and utilizing positive parenting techniques. For a detailed description of the Family Check‐Up, see Dishion et al. (2008).

Of the 731 families (49% females), 272 (37%) were recruited in Pittsburgh, 271 (37%) in Eugene, and 188 (26%) in Charlottesville. Across sites, children were reported to belong to the following racial groups: 27.9% African American, 50.1% European American, 13.0% biracial, and 8.9% other races (e.g., American Indian, Native Hawaiian). In terms of ethnicity, 13.4% reported being Hispanic American. The current study utilized data from a subset of families that participated in Waves 1–9 (data collected between 2002 and 2017) at ages 2, 3, 4, 5, 7.5, 8.5, 9.5, 10.5, and 14 years. A Certificate of Confidentiality was obtained from the National Institute of Health to offer further protection of participants' confidentiality and encourage honest reporting. Institutional Review Board approval was obtained at each site for all screening and assessment procedures.

### Measures

#### Demographic variables

An in‐person semistructured interview was administered at each assessment to the PC to assess demographic characteristics of the family, such as gender, race and ethnicity, household annual income, parent education, and household composition in relation to the TC. For the present study, demographic information on gender, race and ethnicity, site location, and intervention status was collected at the age 2 years assessment, and information on household annual income and parent education was collected at age 10.5 and 14 years and averaged in the analyses to account for change from middle childhood to adolescence. Family contact with child protective services was also assessed at age 14 years during the PC interview.

#### Family structure

Using a similar approach to previous researchers utilizing large, longitudinal data sets of low‐income families (Lee & McLanahan, 2015), we coded family structures based on the relationship between the TC and the adults living in the home and the relationship between the TC and the lists of live‐in caregivers as reported by the PC at TC age 2, 3, 4, 5, 7.5, 8.5, 9.5, 10.5, and 14 years assessments. To extend prior work, which has typically focused on only three family structures (e.g., married parents, non‐married cohabitating parents, and single parents), we emphasized PC relationship to the child and consistency of PC status instead of solely focusing on the PC's romantic relationship status to identify family structures. Further, in light of previous work on intergenerational family structures (DeLeire & Kalil, 2002), we also emphasized the presence of other relatives living in the home and contributing to child‐rearing in identification of family structures. Detailed information on the coding process for identifying family structures present in the data set and power analyses to determine whether the sample sizes in each family composition were large enough to detect an effect are provided in the supplemental materials.

The seven family structures are referred to as Family Composition 1–7. Family structures ranged from most stable to least stable presence of live‐in caregivers. Family Composition 1 is characterized by having the same PC and the same alternate caregiver at all participating waves of the study and both caregivers were in a romantic relationship with one another (e.g., married biological parents of TC). Family Composition 2 was characterized by having the same PC and alternate caregiver at all participating waves of the study and the caregivers were not in a romantic relationship with one another (e.g., biological mother and biological grandmother of TC). Family Composition 3 was defined as having the same PC and grandparent living in the home as well as varying romantic partners of the PC that live in the TC's home. Family Composition 4 was composed of the same PC and varying live‐in relatives of the PC but no live‐in romantic partners (e.g., mother and aunt of TC). Family Composition 5 was defined as having the same PC with varying live‐in romantic partners but no live‐in relatives. Family Composition 6 was composed of the same PC along with varying romantic partners and varying relatives of the PC (e.g., the mother of the TC and her romantic partner living in the TC's home along with the TC's grandmother). Family Composition 7 was characterized by having varying PCs in the participating waves of the study (e.g., biological mother loses custody of the TC and grandmother becomes PC for subsequent waves of the study).

We identified seven family structures that best represented the data, but it is possible that a number of additional compositions of family structure within each of the seven groups could be evident. For example, it was beyond the scope of the current study to investigate caregivers that did not live in the home and, thus, only caregivers living in the home were accounted for in the coding of family structure composition. Accordingly, to reduce the number of groups to a reasonable range for analyzing the data we limited the number to seven family structures that were well‐represented in the sample.

Of the 731 families in the Early Steps Multisite project, we were able to code for family structure using the demographic data for 714 families. Seventeen families did not participate in the demographic interview in at least four waves following the initial age 2 years home visit and thus were not included in the current study. Attrition analyses revealed that the retained families did not differ from attrited families on annual family income or treatment status, (*p* > .05); however, PCs in the attrited families had, on average, lower educational attainment than PCs in the retained sample, *t*(729) = −2.45, *p* = .015.

#### Teacher and primary caregiver reports of middle‐childhood and adolescent disruptive and internalizing problem behaviors

The Teacher Report Form (TRF; Achenbach, 1991) was completed about the TCs by teachers at ages 10.5 and 14 years. The TC identified the teacher that “knew them best” to be contacted by the researchers. On the TRF, teachers rate the child's academic performance and adaptive functioning including the child's behavior. The TRF has 93 items in common with the Child Behavior Checklist (CBCL) version for children age 6–18 years, including all the items on the broadband externalizing and internalizing factors. We utilized the broadband externalizing factor for the TRF at the age 10.5 and 14 years assessments (α = .94 and .88, respectively), which includes the rule‐breaking and aggressive behaviors subscales. The same broadband externalizing factor was used for PC reports on the CBCL at the age 10.5 and 14 assessments (α = .94 and .82, respectively). For TRF internalizing problem behaviors, we utilized the broadband internalizing factor at ages 10.5 and 14 years assessment (α = .83 and .88, respectively). The broadband internalizing factor includes the anxious depressed, withdrawn depressed, and somatic symptom subscales. The same broadband internalizing factor was utilized for PC reports on the CBCL at age 10.5 and 14 years assessments (α = .88 and .90, respectively).

#### Self‐reports of middle childhood and adolescent disruptive problem behavior

Target children completed an adapted version of the Adolescent Self‐report of Deviancy Scale (SRD; Elliot et al., 1985) at the age 10.5 and 14 years assessments (α = .67 and .85, respectively). At age 10.5 years, TCs completed the 27‐item SRD and, at age 14 years, TCs completed the 46‐item SRD, both of which assess the frequency of aggressive and delinquent behaviors an individual has engaged in over the past year. Responses are coded on a Likert scale of 0 (*never*), 1 (*once/twice*), and 2 (*more often*). Sample items include the following: “Have you cheated on school tests? Have you stolen or tried to steal a bicycle or skateboard?”

#### Self‐reports of middle‐childhood internalizing problem behavior

Target children were interviewed by examiners at the age 10.5 assessment utilizing the computer version of the Diagnostic Interview Schedule for Children, Version IV (DISC‐IV‐CR). The DISC‐IV‐CR is a structured diagnostic instrument that assesses 34 common psychiatric diagnoses of children and adolescents based on the *Diagnostic and Statistical Manual of Mental Disorders* (4th ed.; *DSM‐IV*) diagnoses and related symptoms (Shaffer et al., 2000). We utilized total scores on the Generalized Anxiety Disorder and Major Depressive Episode/Dysthymic Disorder symptoms in the current study.

#### Self‐reports of adolescent internalizing problem behavior

The TCs completed an adapted 10‐item version of the Child Depression Inventory (CDI) at the age 14 years assessment (α = .88). The CDI is a measure of depression and is based on the Beck Depression Inventory (BDI; Kovacs & Beck, 1977). Response options were on a 3‐point scale in which a 0 represented *no symptoms*, 1 represented *some symptoms* and 2 represented *symptomatic* responses. A sample item includes 0 = *I do not feel alone*, 1 = *I feel alone many times* and 2 = *I feel alone all the time*.

#### Parent–child relationship quality

The PCs completed the Adult Child Relationship Scale (ACRS) at the ages 2, 5, 9.5, 10.5, and 14 years assessments. The ACRS is a 15‐item measure adapted from the Student‐Teacher Relationship Scale (Pianta et al., 1995), which assesses the quality of the parent–child relationship and yields two factors: Openness/Warmth (five items reverse scored) and Conflict (10 items). A sample item from the Openness/Warmth scale is “If upset, this child seeks comfort from me.” A sample item from the Conflict scale is “This child and I always seem to be struggling with each other.” Responses were rated on a Likert scale of 0 (*definitely not*) to 4 (*definitely*). Primary caregivers' total scores for Openness/Warmth (reverse scored) and Conflict (αs ranged from .65 to .79 at ages 2 to 14 years) were created at each wave of the study and then summed across the five waves to create a composite factor of parent–child relationship quality for the present study. The composite scores at each wave were significantly correlated with one another (*r*s ranging from .26 to .77) with the greatest strength of the association at more proximal waves. Total scores across the five waves for the Openness/Warmth scale and the Conflict scale were significantly correlated with one another (*r* = .40). As the total score for parent–child relationship quality included a composite of Conflict and Openness/Warmth reverse scored, lower total scores represent a more positive relationship quality characterized by lower levels of conflict and higher levels of warmth, support, and communication.

### Data analysis

#### Power analysis

Sample size calculations were conducted to determine whether the sample sizes in each family composition were sufficiently large to detect an effect and were chosen over post hoc power analyses based on recent research suggesting that the observed estimate and significance level highly influence the power level creating biased results (Aberson, 2019; Zhang et al., 2019). Pre‐study power analyses (i.e., sample size calculations) rely on findings from previous research to determine expected effect sizes, which are incorporated into the calculations. A review of the literature provides evidence for an expected effect size in association between family structure, family process, and adolescent adjustment between that of .31 and .33 (see Amato, 2001, for meta‐analysis and Hadfield et al., 2018 for systematic review). Thus, with seven identified family structure types, a significance level of .05, a power level threshold of at least .80, and the effect size range from .31 to .33, a sample size of between 19 and 21 families in each family structure type would be needed. As 30 families is the smallest sample size across the seven identified family structure types, the analyses were adequately powered to detect effects.

#### Analytic strategy

To test our first set of research questions of whether there were similarities in teacher‐reported adolescent disruptive and internalizing problem behavior across the seven identified family structures, we conducted analysis of variance tests (ANOVAs). For our second set of research questions, we assessed whether an index of family *process*, in the form of parent–child openness/warmth and conflict, predicted levels of adolescent disruptive and internalizing problem behavior. To this end, we utilized regression analyses with the current study's full sample and accounted for family structure type. To account for family structure itself in regression analyses, we utilized the contrast codes of −3 to 3 in the regression analysis. Specifically, we used the code −3 = *Family Composition 6*, −2 = *Family Composition 5*, −1 = *Family Composition 4*, 0 = *Family Composition 1*, 1 = *Family Composition 2*, 2 = *Family Composition 7*, and 3 = *Family Composition 3*.

#### Diagnostic assessments of key variables

The primary outcomes of interest (i.e., youth internalizing and disruptive problem behavior) were not normally distributed. To satisfy model assumptions for the analytic strategies described above, we utilized data transformations described below. We chose data transformation rather than using robust standard errors or other approaches, in line with recent work by Knief and Forstmeier (2021), which suggests that this method may be the most helpful approach when variance strongly increases with the mean, as was the case with these data.

##### Teacher and primary caregiver reports of middle‐childhood and adolescent internalizing and disruptive problem behaviors

The teacher‐reported externalizing broadband scores for the age 14 assessment were non‐normally distributed with a skewness of .50 (*SE* = .11) and kurtosis of −.30 (*SE* = .22). A square root transformation was utilized to normalize the data, resulting in a skewness of .33 (*SE* = .11) and kurtosis of −.65 (*SE* = .22), to meet the statistical assumptions of the analyses. The same square root transformation was used for the age 10.5 externalizing broadband scores, age 10.5 internalizing broadband scores, and age 14 internalizing broadband scores, which were all normally distributed, to match scales to ease comparison across the different waves of data collection and types of problem behaviors. The transformations resulted in a skewness of −.10 (*SE* = .10) and kurtosis −.25 (*SE* = .21), a skewness of .05 (*SE* = .14) and kurtosis of −.46 (*SE* = .29), and a skewness of −.06 (*SE* = .11) and kurtosis of −.57 (*SE* = .22), respectively. Additionally, for the same aforementioned reasons, the normally distributed PC‐reported middle‐childhood and adolescent disruptive behavior scores were transformed, which resulted in a skewness of −.21 (*SE* = .10) and kurtosis of −.42 (*SE* = .21), as well as a skewness of −.17 (*SE* = .11) and kurtosis of −.64 (*SE* = .21), respectively. Further, the normally distributed PC‐reported internalizing problem behavior scores at the age 10.5 and 14 assessments were transformed which resulted in a skewness of −.14 (*SE* = .10) and kurtosis of −.76 (*SE* = .21) as well as a skewness of −.18 (*SE* = .10) and kurtosis of −.85 (*SE* = .21), respectively.

##### Adolescent self‐reported middle‐childhood and adolescent disruptive problem behaviors

The age 10.5 and 14 years assessment reports on the Self‐report of Deviancy Scale (SRD) were non‐normally distributed with a skewness of 3.31 (*SE* = .11) and kurtosis of 20.97 (*SE* = .22) and a skewness of 2.84 (*SE* = .11) and kurtosis of 12.50 (*SE* = .21), respectively. An inverse square root transformation was utilized which resulted in skewness of −.05 (*SE* = .11) and kurtosis of −1.39 (SE = .22) and a skewness of .42 (*SE* = .11) and kurtosis of −1.02 (*SE* = .21), respectively.

##### Adolescent self‐reported internalizing problem behaviors

The age 14 years assessment report on the Child Depression Inventory (CDI) was non‐normally distributed with a skewness of 1.37 (*SE* = .11) and kurtosis of .98 (*SE* = .22). A square root transformation was utilized which resulted in a skewness of .38 (*SE* = .11) and kurtosis of −.99 (*SE* = .22).

#### Missing data

Seven hundred thirty‐one families initially participated in the Early Steps Multisite project at the first wave of data collection. An approximately 80% retention rate was achieved across nine waves of data collection. To account for missing data over the nine waves and code for family structure over the waves of data collection, families were included in the subsample if they participated in at least four of the nine waves of data collection from TC age 2 to 14 years. Seven hundred fourteen families participated in at least four waves. To be retained in the present study analytic subsample, families had to participate at both the age 10.5 and 14 years assessments and have participating reporters (e.g., PC, teacher, and TC) for each of the key outcome variables. For longitudinally assessed variables that were averaged (e.g., annual family income and PC education level), if only one wave of data was available it was utilized to maximize sample size. Although the analytic subsample size varied by reporter and by outcome variable, attrition analyses revealed no significant differences on key demographic variables such as gender and treatment status, all *p*s > .05. Specifically, the analytic subsample sizes for disruptive behaviors including all covariates were 435 for teacher reports, 437 for self‐reports, and 470 for PC reports, and the analytic subsample sizes for internalizing behaviors including all covariates were 465 for teacher reports at 14 years but 228 with age 10.5 years teacher reports, 402 for self‐ reports, and 470 for PC reports.

## RESULTS

**FIGURE 1:**
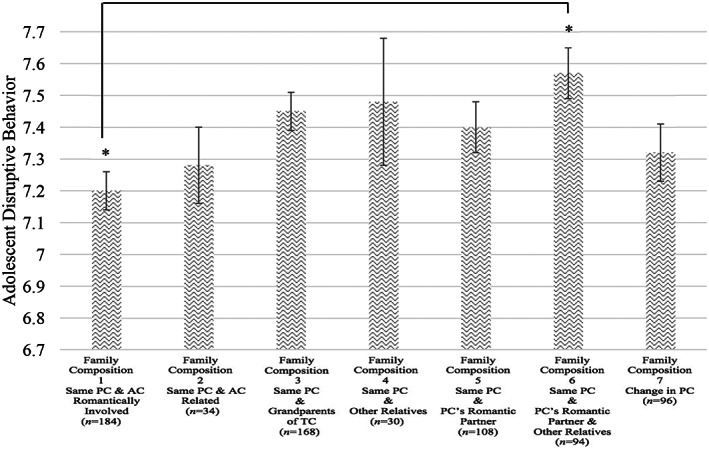
Teacher‐reported adolescent disruptive behavior across family structure types *Note*. AC = alternate caregiver; PC = primary caregiver. Mean scores before accounting for covariates are presented. There were no significant differences after accounting for covariates. *Indicates significant difference at *p* < .05 level in Scheffe post hoc comparison.

Descriptive statistics for each of the seven family structure compositions are reported in Table 1 for categorical variables (e.g., TC gender, site location) and Table 2 for continuous variables (e.g., annual family income). For teacher‐reported adolescent disruptive behavior, the results of the initial univariate ANOVA revealed a significant difference among family structure types for adolescent disruptive problem behavior, *F*(6, 484) = 2.86, *p* = .01. However, Scheffe post hoc comparisons, which can be used when there are differences in sample size across compared groups (Klockars & Sax, 1986), revealed only one significant pairwise difference among the 42 tests computed—a lower percentage of significant differences (2.4%) than would be expected by chance. The difference was between Family Composition 1 (the same PC and AC at all waves who were romantically involved) and Family Composition 6 (the same PC at all waves but varying live‐in relatives and varying PC romantic partners), with lower adolescent disruptive behavior for youth living in stable two‐parent families. See Figure 1 for a depiction of results. However, after accounting for annual family income, PC education, TC gender, TC race, family contact with child protective services, site location of the family, intervention status, and teacher‐reported disruptive behavior at TC age 10.5 years, across the seven family structures there were no significant differences in teacher‐reported adolescent disruptive behavior, *F*(14, 420) = 1.38, *p* = .22. As for teacher‐reported internalizing problem behavior, the results of the initial univariate ANOVA did not reveal a significant difference among family structure types for adolescent internalizing problems, *F*(6, 476) = 1.60, *p* = .15. The results remained nonsignificant when accounting for the aforementioned child, family, and community factors and proximal teacher reports of middle‐childhood internalizing behavior, *F*(14, 213) = 2.10, *p* = .06.

**TABLE 1:** Descriptive statistics of categorical measures

	Family Composition 1: Same PC & AC romantically involved (*n* = 184)		Family Composition 2: Same PC & AC related (*n* = 34)		Family Composition 3: Same PC & grandparents of TC (*n* = 168)		Family Composition 4: Same PC & other relatives (*n* = 30)		Family Composition 5: Same PC & PC's romantic partner (*n* = 108)		Family Composition 6: Same PC & PC's romantic partner & other relatives (*n* = 94)		Family Composition 7: Change in PC (*n* = 96)	
	*N*	%	*N*	%	*N*	%	*N*	%	*N*	%	*N*	%	*N*	%
Intervention status														
Treatment group	88	47.80	17	50.00	85	50.60	11	36.70	56	51.90	53	56.40	48	50.00
Control group	96	52.20	17	50.00	83	49.40	19	63.30	52	48.10	41	43.60	48	50.00
Child gender														
Male	96	52.20	17	50.00	82	48.80	16	53.30	52	48.10	49	52.10	53	55.20
Female	88	47.80	17	50.00	86	51.20	14	46.70	56	51.90	45	47.90	43	44.80
Site location														
Charlottesville, VA	43	23.40	6	17.60	44	26.20	7	23.30	33	30.60	23	24.50	30	31.30
Eugene, OR	91	49.50	18	52.90	46	27.40	9	30.00	34	31.50	33	35.10	37	38.50
Pittsburgh, PA	50	27.20	10	29.40	78	46.40	14	46.70	41	38.00	38	40.40	29	30.20
Contact with child protective services														
Yes	10	6.90	2	5.90	14	10.00	1	7.70	7	7.80	11	14.10	8	11.00
No	135	93.10	32	94.10	126	90.00	12	92.30	83	92.20	67	85.90	65	89.00
PC education level (TC age 14)														
Seventh or less	6	4.10	2	5.90	1	0.70	1	7.70	1	1.10	4	5.10	0	0
Junior high	4	2.70	0	0.00	1	0.70	0	0	1	1.10	0	0	3	4.10
Partial high school	9	6.20	1	2.90	19	13.60	0	0	10	11.10	12	15.40	9	12.20
High school/GED	47	32.20	10	29.40	41	29.30	2	15.40	26	28.90	20	25.60	24	32.40
Partial college	30	20.50	6	17.60	29	20.70	2	15.40	15	16.70	13	16.70	20	27.00
Specialized training	8	5.50	4	11.80	10	7.10	4	30.80	5	5.60	4	5.10	6	8.10
Junior college/associates	30	20.50	8	23.50	28	20.00	4	30.80	23	25.60	15	19.20	6	8.10
Standard college	11	7.50	1	2.90	6	4.30	0	0	6	6.70	9	11.50	3	4.10
Graduate/professional training	1	0.70	2	5.90	5	3.60	0	0	3	3.30	1	1.30	3	4.10

*Note*. AC = alternate caregiver; PC = primary caregiver; TC = target child.

**TABLE 2:** Descriptive statistics of continuous measures

	Family Composition 1: Same PC & AC romantically involved (*n* = 184)		Family Composition 2: Same PC & AC related (*n* = 34)		Family Composition 3: Same PC & grandparents of TC (*n* = 168)		Family Composition 4: Same PC & other relatives (*n* = 30)		Family Composition 5: Same PC & PC's romantic partner (*n* = 108)		Family Composition 6: Same PC & PC's romantic partner & other relatives (*n* = 94)		Family Composition 7: Change in PC (*n* = 96)	
	Mean	*SD*	Mean	*SD*	Mean	*SD*	Mean	*SD*	Mean	*SD*	Mean	*SD*	Mean	*SD*
Average PC years of education (TC age 10.5–14)	12.72	2.34	13.06	2.53	12.93	1.81	13.42	3.48	13.22	1.92	12.56	2.52	12.76	2.09
Average annual family income (TC age 10.5–14)	$42,002	$23,695	$23,026	$14,349	$28,645	$14,348	$16,023	$6,556	$29,540	$17,995	$31,044	$18,894	$35,290	$22,262
Teacher‐reported disruptive behavior (TC age 10.5)	7.26	0.59	7.50	0.64	7.36	0.63	7.46	0.51	7.35	0.69	7.53	0.64	7.31	0.65
Teacher‐reported disruptive behavior (TC age 14)	7.20^a^	0.62	7.28	0.73	7.45	0.70	7.48	0.89	7.40	0.68	7.57 ^a^	0.67	7.32	0.63
Teacher‐reported internalizing behavior (TC age 10.5)	7.33	0.69	7.64	0.51	7.38	0.80	7.01	0.51	7.35	0.71	7.35	0.72	7.49	0.69
Teacher‐reported internalizing behavior (TC age 14)	7.23	0.61	7.49	0.68	7.30	0.66	7.43	0.53	7.48	0.68	7.28	0.71	7.33	0.62
Caregiver‐reported disruptive behavior (TC age 10.5)	7.33	0.77	7.52	0.80	7.41	0.81	7.34	0.96	7.38	0.87	7.54	0.75	7.42	0.72
Caregiver‐reported disruptive behavior (TC age 14)	7.29	0.75	7.15	0.83	7.31	0.85	7.31	0.75	7.28	0.83	7.53	0.80	7.36	0.74
Caregiver‐reported internalizing behavior (TC age 10.5)	7.21	0.76	7.42	0.92	7.22	0.80	7.53	0.95	7.19	0.90	7.27	0.80	7.35	0.85
Caregiver‐reported internalizing behavior (TC age 14)	7.34	0.76	7.53	0.83	7.21	0.86	7.57	0.74	7.17	0.86	7.42	0.87	7.46	0.77
Self‐reported disruptive behavior (TC age 10.5)	0.75	0.22	0.67	0.22	0.72	0.24	0.78	0.25	0.71	0.25	0.74	0.23	0.72	0.24
Self‐reported disruptive behavior (TC age 14)	0.65	0.24	0.60	0.24	0.62	0.26	0.65	0.27	0.57	0.24	0.58	0.27	0.58	0.23
Self‐reported internalizing behavior (TC age 10.5)	0.07	0.33	0.27	0.63	0.08	0.33	0.00	0.00	0.08	0.27	0.10	0.35	0.11	0.50
Self‐reported internalizing behavior (TC age 14)	0.49	0.44	0.59	0.46	0.45	0.44	0.52	0.46	0.57	0.44	0.44	0.42	0.52	0.47
Parent–child relationship quality (TC ages 2, 5, 9.5, 10.5, & 14)	165.11	42.68	173.03	41.74	167.33	41.46	157.88	42.73	159.88	38.49	176.67	45.31	171.00	37.91

*Note*. AC = alternate caregiver; PC = primary caregiver; TC = target child. Parent–child relationship quality is coded such that lower scores indicate a more positive relationship characterized by lower levels of conflict and higher levels of warmth, communication, and support, and higher scores indicate a less positive relationship characterized by higher levels of conflict and lower levels of warmth, communication, and support. Target child self‐reported disruptive behaviors were inverse square root transformed, which resulted in higher scores indicating lower levels of adolescent self‐reported disruptive behaviors. Target child disruptive and internalizing behavior mean scores and parent–child relationship quality mean scores are from the initial univariate analysis of variance tests (ANOVAs) prior to accounting for annual family income, primary caregiver education, target child gender, target child race, family contact with child protective services, site location of the family, intervention status, and proximal reports of child behavior in middle childhood.

^a^ Scheffe post hoc comparisons revealed significant differences for only one pairwise comparison, Family Composition 1 and Family Composition 6, with a mean difference of .37 and standard error of .10, *p* = .01.

Adolescent self‐reports of disruptive and internalizing behaviors converged with the teacher‐reported findings. Specifically, the results of the initial univariate ANOVA did not reveal a significant difference among family structure types for adolescent disruptive behavior problems, *F*(6, 531) = 1.34, *p* = .23. Similar to teacher reports, the results remained nonsignificant for change in self‐reported disruptive behaviors from middle childhood to adolescence when accounting for the aforementioned child, family, and community factors, *F*(14, 422) = 1.38, *p* = .22. As for adolescent self‐reported internalizing behaviors, the initial univariate ANOVA did not reveal a significant difference among family structure types for adolescent internalizing problems, *F*(6, 506) = 0.90, *p* = .50, and remained nonsignificant for change in self‐reported internalizing problems from middle childhood to adolescence when accounting for child, family, and community factors, *F*(14, 387) = 0.88, *p* = .51.

The same pattern emerged for PC‐reported adolescent disruptive and internalizing problem behaviors such that there were no significant differences (*p*s > .05) across family structure types for either type of problem behavior. Similarly, the results remained nonsignificant for change in problem behavior from middle childhood to adolescence and when accounting for child, family, and community factors with the PC reports of disruptive behavior, *F*(14, 455) = 1.79, *p* = .10, and with PC reports of internalizing problem behavior, *F*(14, 455) = 1.88, *p* = .08.

To investigate whether an index of family *process*, in the form of parent–child openness/warmth and conflict, was related to adolescent disruptive and internalizing problem behavior, we first examined such associations utilizing the current study's full sample, regardless of family structure type, in a regression analysis. Parent–child relationship quality significantly predicted teacher‐reported adolescent disruptive behavior at age 14, *R* ^2^ = .06, *F*(1, 368) = 24.26, *p* < .001. For every 1 standardized unit decrease in total scores (reverse coded) for conflict and openness/warmth (i.e., decreased conflict and increased warmth, support, and communication), there was a predicted .25 standardized unit decrease in teacher‐reported adolescent disruptive problem behavior. Hence, as quality of the parent–child relationship increased (i.e., lower levels of conflict and higher levels of warmth, support, and communication), teacher reports of disruptive problem behaviors at age 14 decreased. After accounting for the same aforementioned child, family, and community factors examining associations between family structure type and adolescent disruptive behavior as well as family structure itself, results remained significant, *R* ^2^ = .12, *F*(9, 353) = 5.17, *p* < .001 (see Supplemental Table 4).

However, parent–child relationship quality did not significantly predict *change* in teacher‐reported disruptive behavior from middle childhood to adolescence and neither did other child, family, or community factors including family structure type itself, except for average family income from middle childhood to adolescence. As for internalizing problem behavior, parent–child relationship quality also significantly predicted teacher‐reported adolescent internalizing problem behavior, *R* ^2^ = .07, *F*(1, 361) = 27.01, *p* < .001, and continued to be a significant predictor of change in teacher‐reported internalizing problems from middle childhood to adolescence after accounting for the child, family, and community factors described above including family structure type itself.

Adolescent self‐reports of disruptive and internalizing behaviors again converged with the teacher‐reported findings. Parent–child relationship quality significantly predicted adolescent self‐reported disruptive behavior, *R* ^2^ = .03, *F*(1, 402) = 10.94, *p* = .001, and self‐reported internalizing behavior, *R* ^2^ = .05, *F*(1, 384) = 19.70, *p* < .001. Further, parent–child relationship quality continued to be a significant predictor of change in both adolescent self‐reported disruptive and internalizing behavior from middle childhood to adolescence after accounting for the child, family, and community factors described above including family structure type itself.

To further explore these findings across informant and setting (i.e., at home versus in school), we examined whether parent–child relationship quality predicted PC reports of adolescent disruptive and internalizing behavior. In congruence with teacher reports, we found that PC‐reported adolescent disruptive behavior was significantly predicted by parent–child relationship quality, *R* ^2^ = .40, *F*(1, 410) = 274.11, *p* < .001, which continued to be a significant predictor of change in disruptive behavior from middle childhood to adolescence after accounting for the child, family, and community factors described above including family structure type itself. A similar pattern emerged for the association between parent–child relationship quality and PC‐reported internalizing problem behavior. Parent–child relationship quality significantly predicted PC‐reported adolescent internalizing problem behavior, *R* ^2^ = .22, *F*(1, 412) = 116.36, *p* < .001. Further, parent–child relationship quality continued to be a significant predictor of change in PC‐reported adolescent internalizing problem behavior from middle childhood to adolescence after accounting for the child, family, and community factors described above including family structure type itself. See Table 3 for estimates and significance for problem behavior analyses by all reporters.

**TABLE 3:** Regression analyses

	Parent–child relationship quality ➔ Teacher‐reported adolescent disruptive behavior			Parent–child relationship quality ➔ Teacher‐reported adolescent internalizing behavior			Parent–child relationship quality ➔ Caregiver‐reported adolescent disruptive behavior			Parent–child relationship quality ➔ Caregiver‐reported adolescent internalizing behavior			Parent–child relationship quality ➔ Self‐reported adolescent disruptive behavior			Parent–child relationship quality ➔ Self‐reported adolescent internalizing behavior		
Variable	*β*	*t*	*p*	*β*	*t*	*p*	*β*	*t*	*p*	*β*	*t*	*p*	*β*	*t*	*p*	*β*	*t*	*p*
Parent–child relationship quality (TC ages 2, 5, 9.5, 10.5, & 14)	−.04	−0.64	.52	.16	2.29	.02	.32	6.66	<.001	.20	4.44	<.001	−.10	−1.95	.05	.21	4.05	<.001
Report of TC age 10.5 disruptive behavior	.44	7.12	<.001	.31	4.58	<.001	.45	9.09	<.001	.52	11.61	<.001	.26	5.08	<.001	.14	2.77	.006
Annual family income (TC age 10.5–14)	−.11	−2.03	.04	−.09	−1.23	.22	−.03	−0.90	.37	−.002	−0.05	.96	.04	0.81	.42	−.04	−0.76	.45
Contact with child protective services	−.02	−0.41	.68	.11	1.53	.13	.03	0.80	.42	.01	0.34	.74	.03	0.49	.62	.005	0.09	.93
Intervention status	−.04	−0.92	.36	−.04	−0.63	.53	−.04	−0.98	.33	−.04	−0.98	.33	−.006	−0.13	.90	−.01	−0.24	.81
Site location	.05	1.02	.31	−.006	−0.08	.94	−.02	−0.43	.67	−.006	−0.16	.87	−.09	−1.67	.10	−.05	−1.00	.32
PC education in years (TC age 10.5–14)	−.03	−0.57	.57	−.14	−2.00	.05	−.04	−0.94	.35	−.07	−1.64	.10	−.03	−0.64	.52	.03	0.56	.58
TC gender	−.01	−0.26	.80	−.12	−1.79	.08	.02	0.45	.65	−.07	−1.75	.08	−.08	−1.61	.11	−.32	−6.26	<.001
TC race	−.08	−1.57	.12	.01	0.17	.86	.03	0.93	.35	−.01	−0.24	.81	.005	0.10	.92	.14	2.59	.01
Family composition	−.09	−1.79	.08	.05	0.66	.51	−.02	−0.61	.54	.07	1.86	.06	.02	0.46	.65	.07	1.32	.19

*Note*. TC = target child. Parent–child relationship quality is coded such that lower scores indicate a more positive relationship characterized by lower levels of conflict and higher levels of warmth, communication, and support, and higher scores indicate a less positive relationship characterized by higher levels of conflict and lower levels of warmth, communication, and support. Target child self‐reported disruptive behaviors were inverse square root transformed, which resulted in higher scores indicating lower levels of adolescent self‐reported disruptive behaviors.

## DISCUSSION

Stemming from family privilege in American culture (Letiecq, 2019), there has long been the assumption that children's healthy development is fostered most effectively in two‐married‐parent households compared to other family structure types. Further, based on the additional parenting and financial resources present in two‐parent households, it has been assumed that this pattern would be especially evident in low‐income families. The present study examined whether adolescent disruptive and internalizing behavior were similar across family structures in a large sample of ethnically and racially diverse children in families similarly economically disadvantaged at recruitment.

Bronfenbrenner's ecological systems theory underscores family context as one of the most influential domains of child development (Bronfenbrenner, 1986). In line with recent trends to examine both family structure and family process concurrently (Beckmeyer & Russell, 2018; Crosnoe & Cavanagh, 2010; Murry & Lippold, 2018), the results of the present study are congruent with previous findings that highlight the unique importance of both facets of family context on risk for maladaptive behaviors. The results revealed, across seven modern family structures, no significant differences in self‐reported, teacher‐reported, or PC‐reported disruptive and internalizing problem behavior from middle childhood to adolescence, after accounting for annual family income, PC education, TC gender, TC race, family contact with child protective services, site location of the family, and intervention status. However, family process, in the form of parent–child relationship quality, significantly predicted self‐reported as well as teacher‐ and PC‐reported adolescent disruptive and internalizing problem behavior after accounting for family structure type. Thus, even when accounting for the type of family structure, a positive parent–child relationship, characterized by low levels of conflict and high levels of warmth, support, and communication, was associated with lower levels of adolescent disruptive and internalizing problem behavior.

The results are important for conceptualizing the influence of family context (i.e., family structure and family process) on adolescent problem behaviors for low‐income families from diverse racial and ethnic backgrounds. Adolescence is a time of self‐exploration and identity development (for an overview, see Côté, 2009) and a positive parent–child relationship has been shown to support critical adolescent development (Fuligni & Eccles, 1993). Additionally, the consequences of problem behaviors are exacerbated in adolescence, especially when they manifest in school contexts. Specifically, for disruptive behaviors, acting out or the inability to concentrate can negatively impact adolescent well‐being and academic outcomes (Ackard et al., 2006). In adolescence, academic achievement is critical for educational attainment and has been shown to predict level of success in the transition to adulthood (Deary et al., 2007). As for internalizing problem behaviors, the downstream effects on multiple life domains are substantial. For example, recent research on social withdrawal, which is common with both depressive and anxious symptoms, suggests that withdrawing from social supports and subsequent lack of connection and reduced sense of belonging have profound effects on health (Cole et al., 2015; Leigh‐Hunt et al., 2017) with equal, if not more severe, negative effects to that of daily tobacco smoking on mental and physical health (Holt‐Lunstad et al., 2015). Further, anxious and depressive social withdrawal is linked with increased risk for self‐harm and suicide (Endo et al., 2017). Thus, understanding variables that decrease risk for disruptive problem and internalizing problem behaviors are essential and highlight the need for prevention and intervention techniques that promote family support and positive parent–child relationship quality.

The present study extended prior research by utilizing multiple informants in the longitudinal examination of adolescents' disruptive and internalizing problem behavior across seven modern family structures. A majority of the extant literature has focused on cross‐sectional research or a shorter time period. Thus, by investigating family structure types at repeated waves over a 12‐year period, the present study offered a thorough investigation of the influence of family structures at different developmental time periods on changes in problem behavior during the transition from middle childhood to adolescence. Additionally, the present study incorporated stability into family structure conceptualization and construction whereas prior work has tended to treat family structure as static rather than dynamic. The previous literature also primarily involved studies using three family structures: two biological parents, cohabiting parents, and stepfamilies (Brown, 2004). Our study expanded prior research by including more modern family structures, which allows for more inclusivity for the types of family structures that extend beyond biological/stepparents and are more representative of families in American society today. The sample itself is large and representative of low‐income families from diverse racial and ethnic backgrounds **(**Dishion et al., 2008), whereas most of the prior work concerning adolescent problem behavior across different family structures focused on middle‐ to upper‐class White families (Amato, 2010).

There were a few limitations to the present study that should be considered. In interpreting the findings, we recognized there was potential bias in the reporting of relationship quality, as the PC was the sole reporter of relationship quality. Primary caregivers' perception could be affected by their frame of reference, amount of interaction, and knowledge of the child's life. Importantly, utilizing teachers' reports of adolescent behavior in addition to self‐reported and parent‐reported behavior extends findings from prior work. Future research on parent–child relationship quality could benefit from the use of additional methods such as observational data collection, diaries, and ecological momentary assessments in investigating relationship quality. The inclusion of children with elevated levels of PC‐reported early childhood externalizing problem behavior may limit generalizability. Further, half of the families were randomly assigned to an intervention aimed at increasing positive parenting practices (e.g., positive behavior support, parent involvement), which are well‐known targets for reducing child problem behavior (Dishion et al., 2008). Intervention status was accounted for in all analyses and did not emerge as a significant predictor of adolescent disruptive behavior in the current analysis. Note that reductions in child externalizing problem behavior have been identified for the Family Check‐Up in other publications of the current sample. The reductions found in other publications with this sample were either at earlier waves, such as in early childhood (Dishion et al., 2008; Dishion et al., 2014) and the school‐age years (Dishion et al., 2014; Shaw et al., 2016), or using longitudinal analyses of child problem behavior from ages 2 to 14 (Shaw et al., 2019). An additional limitation was the sociodemographic risk of the sample, as the results may not generalize to higher SES populations, albeit the sample did include a mix of rural, suburban, and urban families. It also remains very important to continue to include participants from low‐income backgrounds to diversify the current scholarship as low‐income participants and participants of color have historically been left out of research literature.

To further extend the findings of the current study, future research should consider the influence of family structures that include caregivers living outside of the home, especially father figures (Cowan & Cowan, 2019). Also, it would be beneficial to examine the influence of sibling relationships as markers of family structure on adolescent internalizing and disruptive behaviors in future research (Sanner & Jensen, 2021). Based on prior work suggesting that the influence of family structure on children's educational outcomes operates differently across various cultural and racial backgrounds of families (Cross, 2020), future research would benefit from between group comparisons in the form of moderation analyses and within group comparisons for families from cultural backgrounds not presently represented in the literature.

Additionally, future research should investigate potential mediating variables that might account for the association between a positive parent–child relationship and adolescent problem behavior, such as parenting stress and resources tied to changes in socioeconomic status. Another potential meditating variable may be physiological reactivity and regulation. Specifically, it may be that parent–child relationship quality and adolescent behavior are linked because a positive and supportive parent–child relationship predicts children's behavioral and physiological regulation (Uchino et al., 1996), which are known protective factors for adolescent problem behaviors (Laursen & Collins, 2009). Another potential mediating candidate may be an epigenetic modification known as methylation. In fact, prior work has shown that harsh parenting techniques significantly predicted methylation of several hypothalamic–pituitary–adrenal axis genes (Lewis et al., 2021), which have been previously linked to child behavior. Further, mother‐reported emotional availability in toddlerhood has been linked to methylation of immune genes in middle childhood, which suggests that the emotional aspect of parent–child relationship quality may influence immune epigenetic profiles with implications for children's health outcomes (Lewis et al., 2020). Similarly, it would benefit future research to incorporate a genetic component to simultaneously consider genetic and family structure and process influences on adolescent problem behaviors (Barnes & Jacobs, 2013). Finally, it is possible that family structure and process interact to influence adolescent internalizing and disruptive behaviors; future research would benefit from examinations that can account for the potential dynamic interplay between family structure and process (Beckmeyer & Russell, 2018; Beckmeyer et al., 2020).

### Implications

Recognizing the meaningful associations between parent–child relationship quality, family structure, and adolescent internalizing and disruptive problem behavior is critical for parents, educators, clinicians, and policy makers. The findings emphasize the need for interventions designed to foster positive parent–child relationships, characterized by low levels of conflict and high levels of warmth, support, and communication. Further, these findings combat stereotypes and stigma typically associated with family structures that deviate from those with the most societal privilege—married parents raising their biological children. The findings suggest that no particular family structure should be incentivized in social and public policy. Instead, policies that inform family resource programming and their funding should strongly focus on promoting caregiver–child relationship quality across family structures by supporting efforts to increase parent warmth, support, and communication. The current findings suggest that these dimensions of the caregiving environment have a positive impact on reductions in adolescent problem behavior. Clinicians working with families should consider these findings in their case conceptualization and development of treatment goals, which should lead them to focus on improving the quality of the parent–child relationship with all caregivers supporting the child's socialization.

## CONCLUSION

The present study revisited the assumption based in family privilege that children develop most adaptively in the nuclear family comprised of married parents raising their children. The study questioned whether children's problem behavior differs across various family structures in a large, ethnically and racially diverse sample of low‐income families. In conclusion, the findings indicated that after accounting for other correlates of poverty, there were no significant differences between varying family structures and adolescent problem behavior. However, including the same conservative use of “third variable” explanatory factors, we found that a positive parent–child relationship quality was associated with reduced adolescent problem behavior even after accounting for family structure type. Overall, the results highlight the importance of a positive parent–child relationship across diverse family structures.

## Supporting information


**Appendix S1** Supporting Information

## References

[fare12728-bib-0001] Aberson, C. L. (2019). Applied power analysis for the behavioral sciences. Routledge.

[fare12728-bib-0002] Achenbach, T. M. (1991). Manual for the Teacher's Report Form and 1991 Profile. University of Vermont: Department of Psychiatry.

[fare12728-bib-0003] Ackard, D. M. , Neumark‐Sztainer, D. , Story, M. , & Perry, C. (2006). Parent–child connectedness and behavioral and emotional health among adolescents. American Journal of Preventive Medicine, 30(1), 59–66. 10.1016/j.amepre.2005.09.013 16414425

[fare12728-bib-0004] Acock, A. C. , & Demo, D. H. (1994). Family diversity and well‐being. Sage.

[fare12728-bib-0005] Amato, P. R. (2001). Children of divorce in the 1990s: An update of the Amato and Keith (1991) meta‐analysis. Journal of Family Psychology, 15(3), 355–370. 10.1037/0893-3200.15.3.355 11584788

[fare12728-bib-0006] Amato, P. R. (1993). Children's adjustment to divorce: Theories, hypotheses, and empirical support. Journal of Marriage and Family, 55(1), 23–38. 10.2307/352954

[fare12728-bib-0007] Amato, P. R. (2010). Research on divorce: Continuing trends and new developments. Journal of Marriage and Family, 72(3), 650–666. 10.1111/j.1741-3737.2010.00723.x

[fare12728-bib-0008] Barnes, J. C. , & Jacobs, B. A. (2013). Genetic risk for violent behavior and environmental exposure to disadvantage and violent crime: The case for gene–environment interaction. Journal of Interpersonal Violence, 28(1), 92–120. 10.1177/0886260512448847 22829212

[fare12728-bib-0009] Beckmeyer, J. J. , & Russell, L. T. (2018). Family structure and family management practices: Associations with positive aspects of youth well‐being. Journal of Family Issues, 39(7), 2131–2154. 10.1177/0192513X17741921

[fare12728-bib-0010] Beckmeyer, J. J. , Su‐Russell, C. , & Russell, L. T. (2020). Family management practices and positive youth development in stepparent and single‐mother families. Family Relations, 69(1), 92–108. 10.1111/fare.12412

[fare12728-bib-0011] Berryhill, M. B. (2016). Mothers' parenting stress and engagement: Mediating role of parental competence. Marriage & Family Review, 52(5), 461–480. 10.1080/01494929.2015.1113600

[fare12728-bib-0012] Bianchi, S. M. , & Casper, L. M. (2000). American families. Population Bulletin, 55, 1–44.

[fare12728-bib-0013] Block, J. H. , Block, J. , & Gjerde, P. F. (1986). The personality of children prior to divorce: A prospective study. Child Development, 57(4), 827–840. 10.2307/1130360 3757603

[fare12728-bib-0014] Branje, S. J. , Hale, W. W., III , Frijns, T. , & Meeus, W. H. (2010). Longitudinal associations between perceived parent‐child relationship quality and depressive symptoms in adolescence. Journal of Abnormal Child Psychology, 38(6), 751–763. 10.1007/s10802-010-9401-6 20217211 PMC2902740

[fare12728-bib-0015] Bronfenbrenner, U. (1986). Ecology of the family as a context for human development: Research perspectives. Developmental Psychology, 22(6), 723–742. 10.1037/0012-1649.22.6.723

[fare12728-bib-0016] Brown, S. L. (2004). Family structure and child well‐being: The significance of parental cohabitation. Journal of Marriage and Family, 66(2), 351–367. 10.1111/j.1741-3737.2004.00025.x

[fare12728-bib-0017] Cherlin, A. J. , Furstenberg, F. F., Jr. , Chase‐Lansdale, L. , Kiernan, K. E. , Robins, P. K. , Morrison, D. R. , & Teitler, J. O. (1991). Longitudinal studies of effects of divorce on children in Great Britain and the United States. Science, 252(5011), 1386–1389. 10.1126/science.2047851 2047851

[fare12728-bib-0018] Cole, S. W. , Capitanio, J. P. , Chun, K. , Arevalo, J. M. , Ma, J. , & Cacioppo, J. T. (2015). Myeloid differentiation architecture of leukocyte transcriptome dynamics in perceived social isolation. Proceedings of the National Academy of Sciences of the United States of America, 112(49), 15142–15147. 10.1073/pnas.1514249112 26598672 PMC4679065

[fare12728-bib-0019] Côté, J. E. (2009). Identity formation and self‐development in adolescence. Handbook of Adolescent Psychology, 1, 266–304.

[fare12728-bib-0020] Cowan, C. P. , & Cowan, P. A. (2019). Enhancing parenting effectiveness, fathers' involvement, couple relationship quality, and children's development: Breaking down silos in family policy making and service delivery. Journal of Family Theory & Review, 11(1), 92–111. 10.1111/jftr.12301

[fare12728-bib-0021] Crosnoe, R. , & Cavanagh, S. E. (2010). Families with children and adolescents: A review, critique, and future agenda. Journal of Marriage and Family, 72(3), 594–611. 10.1111/j.1741-3737.2010.00720.x

[fare12728-bib-0022] Cross, C. J. (2020). Racial/ethnic differences in the association between family structure and children's education. Journal of Marriage and Family, 82(2), 691–712. 10.1111/jomf.12625

[fare12728-bib-0023] Deary, I. J. , Strand, S. , Smith, P. , & Fernandes, C. (2007). Intelligence and educational achievement. Intelligence, 35(1), 13–21. 10.1016/j.intell.2006.02.001

[fare12728-bib-0024] Deleire, T. , & Kalil, A. (2002). Good things come in threes: Single‐parent multigenerational family structure and adolescent adjustment. Demography, 39(2), 393–413. 10.1353/dem.2002.0016 12048958

[fare12728-bib-0025] Demo, D. H. , & Acock, A. C. (1996). Family structure, family process, and adolescent well‐being. Journal of Research on Adolescence, 6, 457–488.

[fare12728-bib-0026] Demuth, S. , & Brown, S. L. (2004). Family structure, family processes, and adolescent delinquency: The significance of parental absence versus parental gender. Journal of Research in Crime and Delinquency, 41(1), 58–81. 10.1177/0022427803256236

[fare12728-bib-0027] Dishion, T. J. , Brennan, L. M. , Shaw, D. S. , McEachern, A. D. , Wilson, M. N. , & Jo, B. (2014). Prevention of problem behavior through annual family check‐ups in early childhood: Intervention effects from home to early elementary school. Journal of Abnormal Child Psychology, 42(3), 343–354. 10.1007/s10802-013-9768-2 24022677 PMC3952033

[fare12728-bib-0028] Dishion, T. J. , Shaw, D. S. , Connell, A. , Gardner, F. , Weaver, C. , & Wilson, M. (2008). The Family Check‐Up with high‐risk indigent families: Preventing problem behavior by increasing parents' positive behavior support in early childhood. Child Development, 79(5), 1395–1414. 10.1111/j.1467-8624.2008.01195.x 18826532 PMC2683384

[fare12728-bib-0029] Dunifon, R. , & Kowaleski‐Jones, L. (2002). Who's in the house? Race differences in cohabitation, single parenthood, and child development. Child Development, 73(4), 1249–1264. 10.1111/1467-8624.00470 12146746

[fare12728-bib-0030] Dunifon, R. , & Kowaleski‐Jones, L. (2007). The influence of grandparents in single‐mother families. Journal of Marriage and Family, 69(2), 465–481. 10.1111/j.1741-3737.2007.00377.x

[fare12728-bib-0031] Elliot, D. S. , Huizinga, D. , & Ageton, S. S. (1985). Explaining delinquency and drug use. Sage.

[fare12728-bib-0032] Emery, R. E. (1982). Interparental conflict and the children of discord and divorce. Psychological Bulletin, 92(2), 310–330. 10.1037/0033-2909.92.2.310 7146231

[fare12728-bib-0033] Endo, K. , Ando, S. , Shimodera, S. , Yamasaki, S. , Usami, S. , Okazaki, Y. , Sasaki, T. , Richards, M. , Hatch, S. , & Nishida, A. (2017). Preference for solitude, social isolation, suicidal ideation, and self‐harm in adolescents. Journal of Adolescent Health, 61(2), 187–191. 10.1016/j.jadohealth.2017.02.018 28457686

[fare12728-bib-0034] Fuligni, A. J. , & Eccles, J. S. (1993). Perceived parent‐child relationships and early adolescents' orientation toward peers. Developmental Psychology, 29(4), 622–632. 10.1037/0012-1649.29.4.622

[fare12728-bib-0035] Ganong, L. , & Coleman, M. (2018). Studying stepfamilies: Four eras of family scholarship. Family Process, 57(1), 7–24. 10.1111/famp.12307 28736896

[fare12728-bib-0036] Ganong, L. , Coleman, M. , & Russell, L. T. (2015). Children in diverse families. In M. H. Bornstein & T. Leventhal (Eds.), Handbook of child psychology and developmental science. Volume 4: Ecological settings and processes in developmental systems (pp. 133–174.). Wiley.

[fare12728-bib-0037] Gray, P. B. , Franco, C. Y. , Garcia, J. R. , Gesselman, A. N. , & Fisher, H. E. (2016). Romantic and dating behaviors among single parents in the United States. Personal Relationships, 23(3), 491–504. 10.1111/pere.12139

[fare12728-bib-0038] Hadfield, K. , Amos, M. , Ungar, M. , Gosselin, J. , & Ganong, L. (2018). Do changes to family structure affect child and family outcomes? A systematic review of the instability hypothesis. Journal of Family Theory & Review, 10(1), 87–110. 10.1111/jftr.12243

[fare12728-bib-0039] Hadfield, K. , & Ungar, M. (2018). Family resilience: Emerging trends in theory and practice. Journal of Family Social Work, 21(2), 81–84. 10.1080/10522158.2018.1424426

[fare12728-bib-0040] Hines, A. M. (1997). Divorce‐related transitions, adolescent development, and the role of the parent‐child relationship: A review of the literature. Journal of Marriage and Family, 59(2), 375–388. 10.2307/353477

[fare12728-bib-0041] Holt‐Lunstad, J. , Smith, T. B. , Baker, M. , Harris, T. , & Stephenson, D. (2015). Loneliness and social isolation as risk factors for mortality: A meta‐analytic review. Perspectives on Psychological Science, 10(2), 227–237. 10.1177/1745691614568352 25910392

[fare12728-bib-0042] Jensen, T. M. (2021). Theorizing ambiguous gain: Opportunities for family scholarship. Journal of Family Theory & Review, 13(1), 100–109. 10.1111/jftr.12401

[fare12728-bib-0043] Kellam, S. G. , Ensminger, M. E. , & Turner, R. J. (1977). Family structure and the mental health of children: Concurrent and longitudinal community‐wide studies. Archives of General Psychiatry, 34(9), 1012–1022. 10.1001/archpsyc.1977.01770210026002 901133

[fare12728-bib-0044] Kennedy, S. , & Bumpass, L. (2008). Cohabitation and children's living arrangements: New estimates from the United States. Demographic Research, 19, 1663–1692. 10.4054/DemRes.2008.19.47 19119426 PMC2612998

[fare12728-bib-0045] Ketsetzis, M. , Ryan, B. A. , & Adams, G. R. (1998). Family processes, parent‐child interactions, and child characteristics influencing school‐based social adjustment. Journal of Marriage and Family, 60(2), 374–387. 10.2307/353855

[fare12728-bib-0046] Klockars, A. J. , & Sax, G. (1986). Post hoc comparisons: The Scheffe test. In A. J. Klockars & G. Sax (Eds.), Multiple comparisons (pp. 59–65). Sage. 10.4135/9781412985185.n4

[fare12728-bib-0047] Knief, U. , & Forstmeier, W. (2021). Violating the normality assumption may be the lesser of two evils. Behavior Research Methods, 53, 2576–2590. 10.3758/s13428-021-01587-5 33963496 PMC8613103

[fare12728-bib-0048] Kovacs, M. , & Beck, A. T. (1977). An empirical‐clinical approach toward a definition of childhood depression. In J. G. Schulterbrandt & A. Raskin (Eds.), Depression in childhood: Diagnosis, treatment, and conceptual models (pp. 1–25). Raven Press.

[fare12728-bib-0049] Krueger, P. M. , Jutte, D. P. , Franzini, L. , Elo, I. , & Hayward, M. D. (2015). Family structure and multiple domains of child well‐being in the United States: A cross‐sectional study. Population Health Metrics, 13(1), Article 6. 10.1186/s12963-015-0038-0 PMC4343278 25729332

[fare12728-bib-0050] Langton, C. E. , & Berger, L. M. (2011). Family structure and adolescent physical health, behavior, and emotional well‐being. The Social Service Review, 85(3), 323–357. 10.1086/661922 23788821 PMC3685438

[fare12728-bib-0051] Laursen, B. , & Collins, W. A. (2009). Parent‐child relationships during adolescence. In R. M. Lerner & L. Steinberg (Eds.), Handbook of adolescent psychology: Contextual influences on adolescent development (pp. 3–42). John Wiley & Sons.

[fare12728-bib-0052] Lee, D. , & McLanahan, S. (2015). Family structure transitions and child development: Instability, selection, and population heterogeneity. American Sociological Review, 80(4), 738–763. 10.1177/0003122415592129 27293242 PMC4902167

[fare12728-bib-0053] Leigh‐Hunt, N. , Bagguley, D. , Bash, K. , Turner, V. , Turnbull, S. , Valtorta, N. , & Caan, W. (2017). An overview of systematic reviews on the public health consequences of social isolation and loneliness. Public Health, 152, 157–171. 10.1016/j.puhe.2017.07.035 28915435

[fare12728-bib-0054] Letiecq, B. L. (2019). Surfacing family privilege and supremacy in family science: Toward justice for all. Journal of Family Theory & Review, 11(3), 398–411. 10.1111/jftr.12338

[fare12728-bib-0055] Lewis, C. R. , Breitenstein, R. S. , Henderson, A. , Sowards, H. A. , Piras, I. S. , Huentelman, M. J. , Doane, L. D. , & Lemery‐Chalfant, K. (2021). Harsh parenting predicts novel HPA receptor gene methylation and NR3C1 methylation predicts cortisol daily slope in middle childhood. Cellular and Molecular Neurobiology, 41(4), 783–793. 10.1007/s10571-020-00885-4 32472381 PMC11448560

[fare12728-bib-0056] Lewis, C. R. , Sowards, H. A. , Huentelman, M. J. , Doane, L. D. , & Lemery‐Chalfant, K. (2020). Epigenetic differences in inflammation genes of monozygotic twins are related to parental emotional availability and health. Brain, Behavior, & Immunity‐Health, 5, Article 100084. 10.1016/j.bbih.2020.100084 PMC8474531 34589859

[fare12728-bib-0057] Magnuson, K. & Berger, L. M. (2009). Family structure states and transitions: Associations with children's well‐being during middle childhood. Journal of Marriage and Family, 71(3), 575–591. 10.1111/j.1741-3737.2009.00620.x 20228952 PMC2836533

[fare12728-bib-0058] Manning, W. D. , & Lamb, K. A. (2003). Adolescent well‐being in cohabiting, married, and single‐parent families. Journal of Marriage and Family, 65(4), 876–893. 10.1111/j.1741-3737.2003.00876.x

[fare12728-bib-0059] Mollborn, S. , Fomby, P. , & Dennis, J. A. (2011). Who matters for children's early development? race/ethnicity and extended household structures in the United States. Child Indicators Research, 4(3), 389–411. 10.1007/s12187-010-9090-2 21927627 PMC3172319

[fare12728-bib-0060] Morrison, E. F. , Rimm‐Kauffman, S. , & Pianta, R. C. (2003). A longitudinal study of mother–child interactions at school entry and social and academic outcomes in middle school. Journal of School Psychology, 41(3), 185–200. 10.1016/S0022-4405(03)00044-X

[fare12728-bib-0061] Murry, V. M. , & Lippold, M. A. (2018). Parenting practices in diverse family structures: Examination of adolescents' development and adjustment. Journal of Research on Adolescence, 28(3), 650–664. 10.1111/jora.12390 30515943

[fare12728-bib-0062] Musick, K. , & Meier, A. (2010). Are both parents always better than one? Parental conflict and young adult well‐being. Social Science Research, 39(5), 814–830. 10.1016/j.ssresearch.2010.03.002 20824195 PMC2930824

[fare12728-bib-0063] Nock, S. L. (2005). Marriage as a public issue. The Future of Children, 15(2), 13–32. 10.1353/foc.2005.0019 16158728

[fare12728-bib-0064] Nuttall, E. V. , & Nuttall, R. L. (1976). Parent‐child relationships and effective academic motivation. The Journal of Psychology, 94(1), 127–133. 10.1080/00223980.1976.9921406

[fare12728-bib-0065] O'Donnell, D. A. , Schwab‐Stone, M. E. , & Muyeed, A. Z. (2002). Multidimensional resilience in urban children exposed to community violence. Child Development, 73(4), 1265–1282. 10.1111/1467-8624.00471 12146747

[fare12728-bib-0066] Pettit, G. S. , Keiley, M. K. , Laird, R. D. , Bates, J. E. , & Dodge, K. A. (2007). Predicting the developmental course of mother‐reported monitoring across childhood and adolescence from early proactive parenting, child temperament, and parents' worries. Journal of Family Psychology, 21(2), 206–217. 10.1037/0893-3200.21.2.206 17605543 PMC2791369

[fare12728-bib-0067] Pianta, R. C. , Steinberg, M. S. , & Rollins, K. B. (1995). The first two years of school: Teacher‐child relationships and deflections in children's classroom adjustment. Development and Psychopathology, 7(2), 295–312. 10.1017/S0954579400006519

[fare12728-bib-0068] Powell, B. , Hamilton, L. , Manago, B. , & Cheng, S. (2016). Implications of changing family forms for children. Annual Review of Sociology, 42, 301–322. 10.1146/annurev-soc-081715-074444

[fare12728-bib-0069] Qu, Y. , Fuligni, A. J. , Galvan, A. , & Telzer, E. H. (2015). Buffering effect of positive parent–child relationships on adolescent risk taking: A longitudinal neuroimaging investigation. Developmental Cognitive Neuroscience, 15, 26–34. 10.1016/j.dcn.2015.08.005 26342184 PMC4639442

[fare12728-bib-0070] Rothbaum, F. , & Weisz, J. R. (1994). Parental caregiving and child externalizing behavior in nonclinical samples: A meta‐analysis. Psychological Bulletin, 116(1), 55–74. 10.1037/0033-2909.116.1.55 8078975

[fare12728-bib-0071] Russell, L. T. , Coleman, M. , & Ganong, L. (2018). Conceptualizing family structure in a social determinants of health framework. Journal of Family Theory & Review, 10(4), 735–748. 10.1111/jftr.12296

[fare12728-bib-0072] Ryan, R. M. , & Claessens, A. (2013). Associations between family structure changes and children's behavior problems: The moderating effects of timing and marital birth. Developmental Psychology, 49(7), 1219–1231. 10.1037/a0029397 22866830

[fare12728-bib-0073] Sanner, C. , & Jensen, T. M. (2021). Toward more accurate measures of family structure: Accounting for sibling complexity. Journal of Family Theory & Review, 13(1), 110–127. 10.1111/jftr.12406

[fare12728-bib-0074] Sarsour, K. , Sheridan, M. , Jutte, D. , Nuru‐Jeter, A. , Hinshaw, S. , & Boyce, W. T. (2011). Family socioeconomic status and child executive functions: The roles of language, home environment, and single parenthood. Journal of the International Neuropsychological Society, 17(1), 120–132. 10.1017/S1355617710001335 21073770

[fare12728-bib-0075] Savell, S. M. , Womack, S. R. , Wilson, M. N. , Shaw, D. S. , & Dishion, T. J. (2019). Considering the role of early discrimination experiences and the parent–child relationship in the development of disruptive behaviors in adolescence. Infant Mental Health Journal, 40(1), 98–112. 10.1002/imhj.21752 30586478 PMC7304493

[fare12728-bib-0076] Shaffer, D. , Fisher, P. , Lucas, C. P. , Dulcan, M. K. , & Schwab‐Stone, M. E. (2000). NIMH Diagnostic Interview Schedule for Children Version IV (NIMH DISC‐IV): Description, differences from previous versions, and reliability of some common diagnoses. Journal of the American Academy of Child & Adolescent Psychiatry, 39(1), 28–38. 10.1097/00004583-200001000-00014 10638065

[fare12728-bib-0077] Shaw, D. S. (1991). The effects of divorce on children's adjustment: Review and implications. Behavior Modification, 15(4), 456–485. 10.1177/01454455910154002 1747089

[fare12728-bib-0078] Shaw, D. S. , Emery, R. E. , & Tuer, M. D. (1993). Parental functioning and children's adjustment in families of divorce: A prospective study. Journal of Abnormal Child Psychology, 21(1), 119–134. 10.1007/BF00910493 8463501

[fare12728-bib-0079] Shaw, D. S. , Galán, C. , Lemery‐Chalfant, K. , Dishion, T. J. , Elam, K. K. , Wilson, M. N. , & Gardner, F. (2019). Trajectories and predictors of children's early‐starting conduct problems: Child, family, genetic, and intervention effects. Development and Psychopathology, 31(5), 1911–1921. 10.1017/S0954579419000828 31370912 PMC7366854

[fare12728-bib-0080] Shaw, D. S. , Sitnick, S. , Brennan, L. M. , Choe, D. E. , Dishion, T. J. , Wilson, M. N. , & Gardner, F. (2016). The long‐term effectiveness of the Family Check‐Up on school‐age conduct problems: Moderation by neighborhood deprivation. Development and Psychopathology, 28(4), 1471–1486. 10.1017/S0954579415001212 26646197 PMC4900930

[fare12728-bib-0081] Shaw, D. S. , Winslow, E. B. , & Flanagan, C. (1999). A prospective study of the effects of marital status and family relations on young children's adjustment among African American and European American families. Child Development, 70(3), 742–755. 10.1111/1467-8624.00053 10368919

[fare12728-bib-0082] Thornton, A. (Ed.). (2001). The well‐being of children and families: Research and data needs. University of Michigan Press. 10.3998/mpub.11436

[fare12728-bib-0083] Uchino, B. N. , Cacioppo, J. T. , & Kiecolt‐Glaser, J. K. (1996). The relationship between social support and physiological processes: A review with emphasis on underlying mechanisms and implications for health. Psychological Bulletin, 119(3), 488–531. 10.1037/0033-2909.119.3.488 8668748

[fare12728-bib-0084] U.S. Census Bureau . (2001). Profiles of general demographic characteristics:2000 Census of population and housing. United States. https://www2.census.gov/library/publications/2001/dec/2kh00.pdf.

[fare12728-bib-0085] Walsh, F. (Ed.). (1982). Normal family processes. Guilford Press.

[fare12728-bib-0086] Zhang, Y. , Hedo, R. , Rivera, A. , Rull, R. , Richardson, S. , & Tu, X. M. (2019). Post hoc power analysis: Is it an informative and meaningful analysis? *General* Psychiatry, 32(4), Article e100069. 10.1136/gpsych-2019-100069 PMC6738696 31552383

