# Supplementary material for: Family processes and structure: Longitudinal influences on adolescent disruptive and internalizing behaviors
Source: Fam Relat. 2022 Jul 6;72(1):361–82. doi: 10.1111/fare.12728 (PMC10084072; doi:10.1111/fare.12728)
Supplement: Supplementary file 1 — Appendix S1 Supporting Information [file FARE-72-361-s001.docx]

**Supplemental Materials**

| **Table 4**  ***Teacher-reported Adolescent Disruptive Behavior***  ***without Proximal Behavior Accounted for*** | | | | | | | | |  |
| --- | --- | --- | --- | --- | --- | --- | --- | --- | --- |
| *Parent-child Relationship*  *Quality* 🡪 *Teacher-reported.*  *Adolescent Disruptive*  *Problem Behavior* | | | | | | | | |  |
| Variable | *β* | *t* | *p* |  | |  |  |  |  |
| Parent-child Relationship  Quality (TC Ages 2, 5, 9.5, 10.5, & 14) | .23 | 4.47 | <.001 | |  | |  |  |  |
| Annual Family Income (TC Age 10-14) | -.15 | -2.67 | .008 | |  | |  |  |  |
| Contact with Child Protective  Services | .001 | -0.01 | .99 | |  | |  |  |  |
| Intervention Status | -.01 | -0.26 | .80 | |  | |  |  |  |
| Site location | .08 | 1.58 | .12 | |  | |  |  |  |
| PC Education in Years  (TC Age 10-14) | -.02 | -0.32 | .75 | |  | |  |  |  |
| TC Gender | .05 | 1.01 | .31 | |  | |  |  |  |
| TC Race | -.06 | -1.11 | .27 | |  | |  |  |  |
| Family Composition | -.08 | -1.59 | .11 | |  | |  |  |  |

*Note.* The parent-child relationship quality was scored such that higher scores indicate a

less positive relationship with higher levels of conflict and lower levels of warmth, support,

and communication.

**Family Structure Coding**

In the family structure coding process, we initially identified families in which the primary and alternate caregivers remained the same across the nine waves of the study based on research indicating the negative impact of family structure transitions on child and adolescent disruptive behavior (Fomby & Sennott, 2013; Womack et al., 2019). Next, utilizing protocols followed by other family structure coding systems (e.g., DeLeire & Kalil, 2002; Kellam et al., 1977; Lee & McLanahan, 2015), we determined whether the primary and alternate caregiver were in a romantic relationship (e.g., could be dating or married) or were relatives (e.g., mother and grandmother of TC). Although previous researchers have found that having two parents, regardless of whether those two parents are married or related, is associated with lower levels of adolescent problem behavior compared to single parent households (Brown, 2004; Kellam et al., 1977; DeLeire & Kalil, 2002), there are mixed findings for whether adolescent behavior differs across types of two-parent households. Then, based on findings that instability in family structure can be linked to higher risk of adolescent problem behavior (Brown, 2004; Demuth & Brown, 2004), we identified families in which the primary caregiver remained the same across the nine waves but the alternate caregiver, primary caregiver’s romantic partner, or other relatives living in the home (e.g., grandparents, cousins) varied over time. Next, within that group of families that had the same primary caregiver across the nine waves of the study, we further differentiated groups by the relationship of the adult caregivers living in the home to the target child (i.e., only the primary caregiver’s romantic partner(s), only the target child’s relatives, or a combination of both the primary caregiver’s romantic partner(s) and the target child’s relatives) in a similar fashion to the approach by DeLeire and Kalil (2002) and Lee and McLanahan (2015). Through emphasizing consistency in caregiver status and relationship to the target child in the methods described above, we identified seven different family structures represented in the data. Using a similar system, DeLeire et al. (2002) identified 10 family structures. However, we identified only seven family structures primarily because of 1) our emphasis on consistency in caregiver status and caregiver relationship to the target child rather than the caregivers’ marital status and 2) the high percentage of female primary caregivers in the study.

**References**

Brown, S. L. (2004). Family structure and child well-being: The significance of parental cohabitation. *Journal of Marriage and Family*, *66*(2), 351–367. https://doi.org/10.1111/j.1741-3737.2004.00025.x

Deleire, T., & Kalil, A. (2002). Good things come in threes: Single-parent multigenerational family structure and adolescent adjustment. *Demography*, *39*(2), 393–413. <https://doi.org/10.1353/dem.2002.0016>

Demuth, S., & Brown, S. L. (2004). Family structure, family processes, and adolescent delinquency: The significance of parental absence versus parental gender. *Journal of Research in Crime and Delinquency*, *41*(1), 58–81. https://doi.org/10.1177/0022427803256236

Fomby, P., & Sennott, C. A. (2013). Family structure instability and mobility: The consequences for adolescents’ problem behavior. *Social Science Research*, *42*(1), 186–201. <https://doi.org/10.1016/j.ssresearch.2012.08.016>

Kellam, S. G., Ensminger, M. E., & Turner, R. J. (1977). Family structure and the mental health of children: Concurrent and longitudinal community-wide studies. *Archives of General Psychiatry*, 34(9), 1012–1022. <https://doi.org/10.1001/archpsyc.1977.01770210026002>

Lee, D., & McLanahan, S. (2015). Family structure transitions and child development: Instability, selection, and population heterogeneity. *American Sociological Review*, *80*(4), 738–763. https://doi.org/10.1177/0003122415592129

Womack, S. R., Taraban, L., Shaw, D. S., Wilson, M. N., & Dishion, T. J. (2019). Family turbulence and child internalizing and externalizing behaviors: Moderation of effects by race. *Child Development*, *90*(6), e729–e744. https://doi.org/10.1111/cdev.13103 PMID:29921025
